# Supplementary material for: Investigating Eating Habits of Children Aged between 6 Months and 3 Years in the Provinces of Modena and Reggio Emilia: Is Our Kids’ Diet Sustainable for Their and the Planet’s Health?
Source: Healthcare (Basel). 2024 Feb 10;12(4):453. doi: 10.3390/healthcare12040453 (PMC10888164; doi:10.3390/healthcare12040453)
Supplement: Supplementary file 1 [file healthcare-12-00453-s001.zip › healthcare-2796127-supplementary.pdf]

Investigating Eating Habits of Children Aged Between 6 Months and 3 Years in The Province of Modena and Reggio Emilia: Is Our Kids Diet Sustainable for Their and the Planet's Health?

Lucia Palandri[1,2*] †, Laura Rocca[3] †, Maria Rosaria Scasserra[1], Giacomo Pietro Vigezzi[4,5], Anna Odone[4], Lorenzo Iughetti[3,6], Laura Lucaccioni[3] and Elena Righi[1]

Supplementary material

Figure S1:

Estimation of of the optimal number of clusters was performed using the Silhouette method for describing adherence clustering for main food categories without accounting for school meals

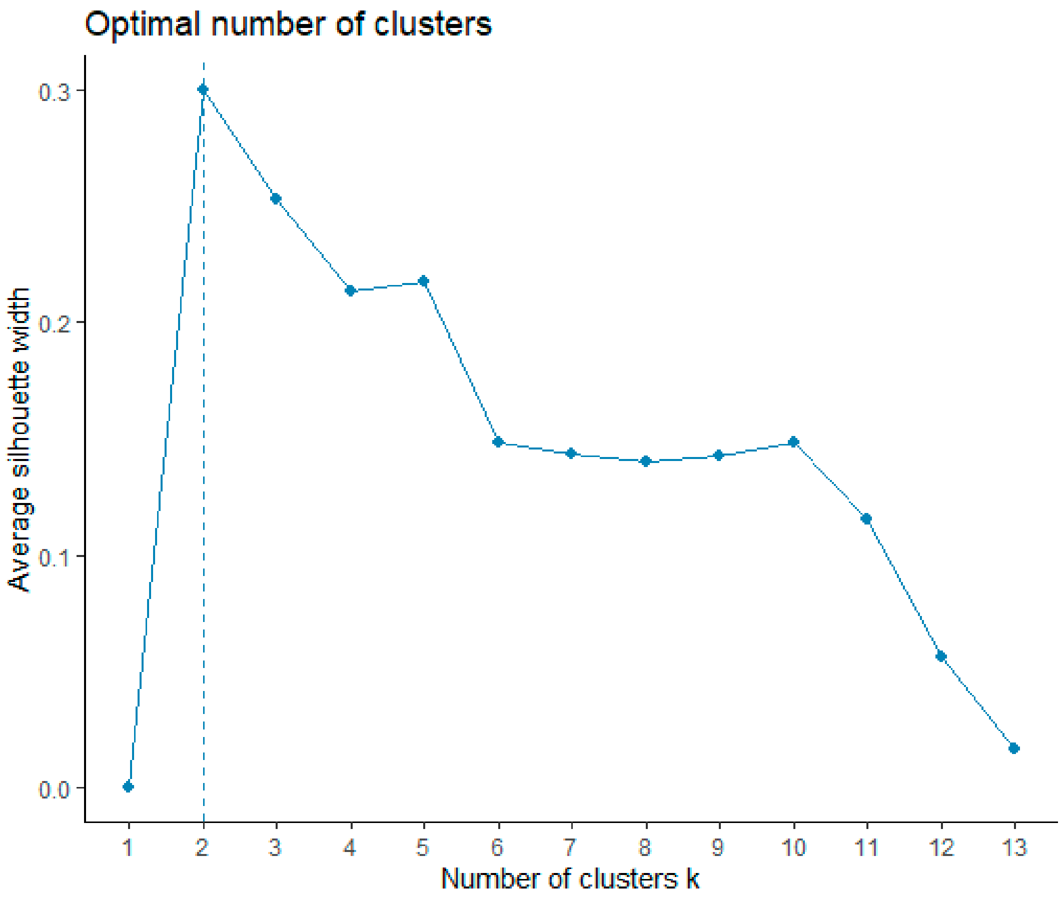

Figure S2:

Estimation of of the optimal number of clusters was performed using the Silhouette method for describing adherence clustering for main food categories without accounting for school meals

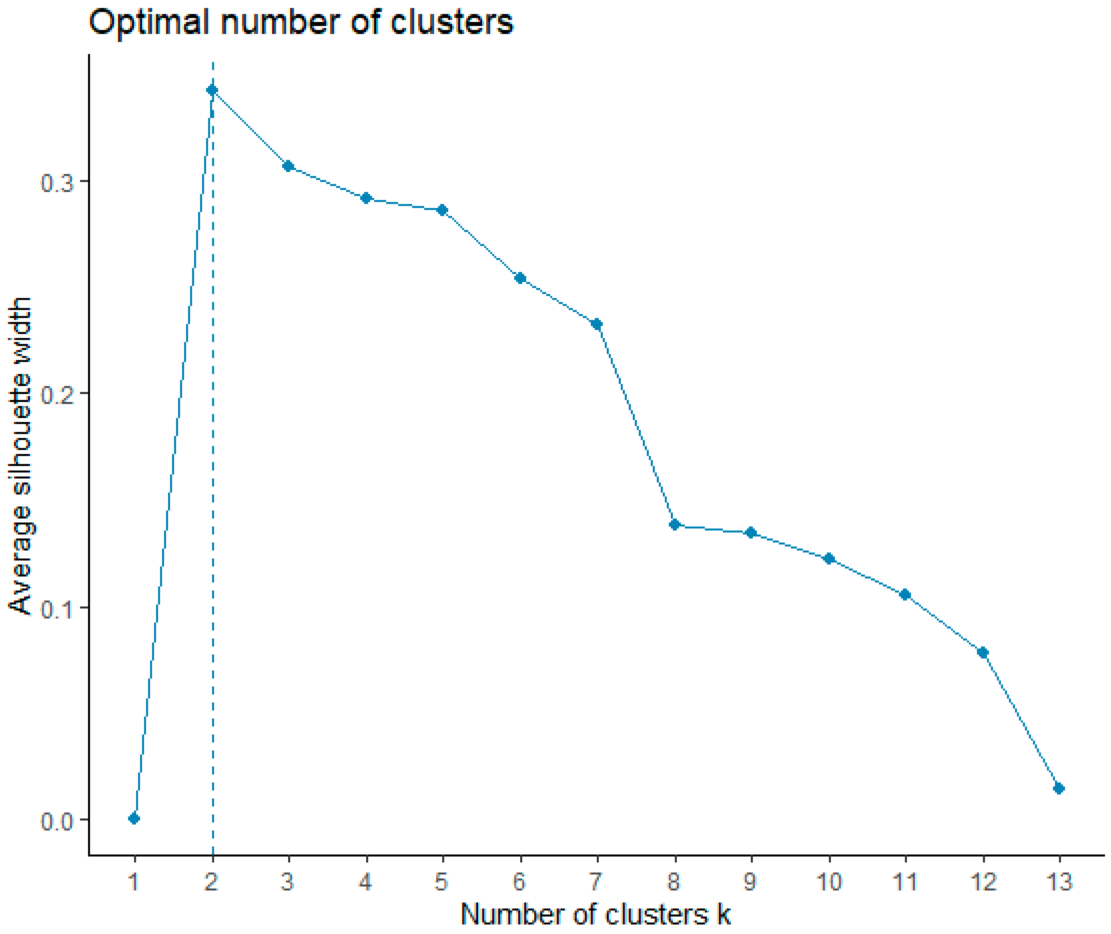

Table S1.

Children's food frequency consumption for all the food categories in the overall sample and divided by age categories. *Total Cereals*: pasta, bread, rice, other cereals (barley, spelt, corn, quinoa, buckweat, oat, kamut), bread substitutes (crackers, breadsticks), breakfast cereals. *Total Meat*: red meat (beef, veal, pork, sheep, goat, game meat, red meat in the pasta sauce), white meat (chicken, turkey, rabbit, white meat in the pasta sauce). *Processed Meat*: cured meat, dried meat, cooked ham, salami, bresaola, sausage, industrial burgers. *Fish*: fresh fish, frozen fish, clams, crustaceans. *Legumes*: beans, peas, chickpeas, lentils, grass peas. *Total Cheese*: light cheese, with less than 25% fat (mozzarella, stracchino, crescenza, ricotta, provola), fatty cheese, with more than 25% fat (robiola, caciotta cheese, aged cheese). *Vegetables:* fresh vegetables, leafy salads (excluding potatoes). *Sweets*: ice cream, pudding, candies, nutella, chocolate, bakery products (cake, tarts, croissants, biscuits). *Sugary Soft Drinks*: fruit juice, orange soda, sweet tea, coke soda. *Processed Food*: sweet snacks, savory snacks, potato chips, ready-to-eat and pre-cooked food, fish sticks, fast food, fruit in syrup.

		Tot = 218	6-1y = 66	2-3 yrs = 152	p-value
Total Cereals					0.004
	2-3 per day	147 (67%)	53 (80%)	94 (62%)	
	1 per day	29 (13%)	10 (15%)	19 (13%)	
	Almost daily	35 (16%)	3 (5%)	32 (21%)	
	3-5 per week	5 (2%)	0 (0%)	5 (3%)	
	2 per week	2 (1%)	0 (0%)	2 (1%)	
**Pasta and Bread**					0.026
	1-2 per day	134 (61%)	48 (73%)	86 (57%)	
	4-6 per week	59 (27%)	15 (23%)	44 (29%)	
	2-3 per week	20 (9%)	2 (3%)	18 (12%)	
	1 per week	1 (2%)	0 (0%)	4 (3%)	
	Rarely/never	1 (1%)	1 (2%)	0 (0%)	
**Rice**					0.2
	1-2 per day	23 (11%)	8 (12%)	15 (10%)	
	4-6 per week	7 (3%)	4 (6%)	3 (2%)	
	2-3 per week	87 (40%)	20 (30%)	67 (44%)	
	1 per week	76 (35%)	25 (38%)	51 (34%)	
	Rarely/never	25 (11%)	9 (14%)	16 (11%)	
**Other Cereals**					0.3
	1-2 per day	18 (8%)	8 (12%)	10 (7%)	
	4-6 per week	11 (5%)	5 (8%)	6 (4%)	
	2-3 per week	65 (30%)	15 (23%)	50 (33%)	
	1 per week	55 (25%)	17 (38%)	38 (25%)	
	Rarely/never	69 (32%)	21 (32%)	48 (32%)	
Total Meat					0.2
	1-2 per day	28 (13%)	12 (18%)	16 (11%)	
	Almost daily	31 (14%)	10 (15%)	21 (14%)	
	4 per week	79 (36%)	23 (35%)	56 (37%)	
	3 per week	37 (17%)	10 (15%)	27 (18%)	
	2 per week	30 (14%)	6 (9%)	24 (16%)	
	1 per week	7 (3%)	1 (2%)	6 (4%)	
	Rarely/never	28 (13%)	12 (18%)	16 (11%)	
**Red Meat**					0.074
	Almost daily	4 (2%)	3 (5%)	1 (1%)	
	4-5 per week	6 (3%)	2 (3%)	4 (3%)	
	2-3 per week	67 (31%)	21 (32%)	46 (30%)	
	1 per week	102 (47%)	24 (36%)	78 (51%)	
	Rarely/never	39 (18%)	16 (24%)	23 (15%)	
**White Meat**					<0.001

	Almost daily	9 (4%)	7 (11%)	2 (1%)	
	4-5 per week	12 (6%)	8 (12%)	4 (3%)	
	2-3 per week	111 (51%)	34 (52%)	77 (51%)	
	1 per week	70 (32%)	12 (18%)	58 (38%)	
	Rarely/never	16 (7%)	5 (8%)	11 (7%)	
Processed Meat					0.004
	Almost daily	3 (1%)	3 (5%)	0 (0%)	
	4-5 per week	5 (2%)	1 (2%)	4 (3%)	
	2-3 per week	21 (10%)	4 (6%)	17 (11%)	
	1 per week	92 (42%)	20 (30%)	72 (47%)	
	Rarely/never	97 (44%)	38 (58%)	59 (39%)	
Fish					0.5
	4-5 per week	8 (4%)	3 (5%)	5 (3%)	
	2-3 per week	107 (49%)	37 (56%)	70 (46%)	
	1 per week	88 (40%)	22 (33%)	66 (43%)	
	Rarely/never	15 (7%)	4 (6%)	11 (7%)	
Legumes					0.001
	Almost daily	11 (5%)	6 (9%)	5 (3%)	
	4-5 per week	25 (11%)	15 (23%)	10 (7%)	
	2-3 per week	108 (50%)	28 (42%)	80 (53%)	
	1 per week	58 (27%)	11 (17%)	47 (31%)	
	Rarely/never	16 (7%)	6 (9%)	10 (7%)	
Total Cheese					0.12
	1 per day	77 (35%)	32 (48%)	45 (30%)	
	Almost daily	34 (16%)	9 (14%)	25 (16%)	
	4-5 per week	13 (6%)	2 (3%)	11 (7%)	
	3-4 per week	42 (19%)	8 (12%)	34 (22%)	
	2-3 per week	27 (12%)	7 (11%)	20 (13%)	
	1-2 per week	25 (11%)	8 (12%)	17 (11%)	
**Light Cheese**					0.14
	Almost daily	3 (1%)	2 (3%)	1 (1%)	
	4-5 per week	6 (3%)	4 (6%)	2 (1%)	
	2-3 per week	57 (26%)	14 (21%)	43 (28%)	
	1 per week	106 (49%)	31 (47%)	75 (49%)	
	Rarely/never	46 (21%)	15 (23%)	31 (20%)	
**Fatty Cheese**					>0.9
	Almost daily	1 (1%)	0 (0%)	1 (1%)	
	4-5 per week	3 (1%)	1 (2%)	2 (1%)	
	2-3 per week	19 (9%)	6 (9%)	13 (9%)	
	1 per week	88 (40%)	26 (39%)	62 (41%)	
	Rarely/never	107 (49%)	33 (50%)	74 (49%)	
**Parmesan Cheese**					0.055
	Almost daily	63 (29%)	27 (41%)	36 (24%)	
	4-5 per week	41 (19%)	14 (21%)	27 (18%)	
	2-3 per week	66 (30%)	13 (20%)	53 (35%)	
	1 per week	38 (17%)	10 (15%)	28 (18%)	
	Rarely/never	10 (5%)	2 (3%)	8 (5%)	
**Melted Cheeses**					0.6
	Almost daily	4 (2%)	2 (3%)	2 (1%)	
	4-5 per week	6 (3%)	2 (3%)	4 (3%)	
	2-3 per week	20 (9%)	5 (8%)	15 (10%)	
	1 per week	40 (18%)	15 (23%)	25 (16%)	
	Rarely/never	148 (68%)	42 (64%)	106 (70%)	

Vegetables					<0.001
	1-2 per day	108 (50%)	48 (73%)	60 (39%)	
	1 per day	39 (18%)	5 (8%)	34 (22%)	
	4-6 per week	42 (19%)	10 (15%)	32 (21%)	
	2-3 per week	20 (9%)	2 (3%)	18 (12%)	
	1 per week	5 (2%)	0 (0%)	5 (3%)	
	Rarely/never	4 (2%)	1 (2%)	3 (2%)	
**Fresh fruit**					0.080
	1-2 per day	121 (56%)	44 (67%)	77 (51%)	
	1 per day	49 (22%)	9 (14%)	40 (26%)	
	4-6 per week	29 (13%)	8 (12%)	21 (14%)	
	2-3 per week	10 (5%)	2 (3%)	8 (5%)	
	1 per week	4 (2%)	0 (0%)	4 (3%)	
	Rarely/never	5 (2%)	3 (5%)	2 (1%)	
**Dried fruit**					0.001
	1-2 per day	5 (2%)	4 (6%)	1 (1%)	
	1 per day	31 (14%)	6 (9%)	25 (16%)	
	4-6 per week	24 (11%)	2 (3%)	22 (14%)	
	2-3 per week	22 (10%)	6 (9%)	16 (11%)	
	Rarely/never	95 (44%)	39 (59%)	56 (37%)	
**Milk (cow)**					<0.001
	> 2 per day	10 (5%)	0 (0%)	10 (7%)	
	1-2 per day	72 (33%)	9 (14%)	63 (41%)	
	4-6 per week	17 (8%)	2 (3%)	15 (10%)	
	2-3 per week	15 (7%)	2 (3%)	13 (9%)	
	1 per week	14 (6%)	3 (5%)	11 (7%)	
	Rarely/never	90 (41%)	50 (76%)	40 (26%)	
**Follow-on milk**					<0.001
	> 2 per day	13 (6%)	9 (14%)	4 (3%)	
	1-2 per day	19 (9%)	11 (17%)	8 (5%)	
	4-6 per week	4 (2%)	1 (2%)	3 (2%)	
	2-3 per week	4 (2%)	2 (3%)	2 (1%)	
	1 per week	2 (1%)	0 (0%)	2 (1%)	
	Rarely/never	176 (81%)	43 (65%)	133 (88%)	
**Yogurt**					0.11
	> 2 per day	4 (2%)	0 (0%)	4 (3%)	
	1-2 per day	28 (13%)	9 (14%)	19 (13%)	
	4-6 per week	40 (18%)	12 (18%)	28(18%)	
	2-3 per week	77 (35%	24 (36%)	53 (35%)	
	1 per week	40 (18%)	7 (11%)	33 (22%)	
	Rarely/never	29 (13%)	14 (21%)	15 (10%)	
**Eggs**					0.3
	Almost daily	1 (1%)	0 (0%)	1 (1%)	
	3-5 eggs per week	2 (1%)	1 (2%)	1 (1%)	
	2 eggs per week	50 (23%)	11 (17%)	39 (26%)	
	1 egg per week	123 (56%)	37 (56%)	86 (57%)	
	Rarely/never	42 (19%)	17 (26%)	25 (16%)	
Sweets					<0.001
	1-2 per day	3 (1%)	0 (0%)	3 (2%)	
	Almost daily	21 (10%)	1 (2%)	20 (13%)	
	3-5 per week	33 (15%)	6 (9%)	27 (18%)	
	1-2 per week	71 (33%)	6 (9%)	65 (43%)	

	Rarely/never	90 (41%)	53 (80%)	37 (24%)	
Sugary Soft Drinks					<0.001
	Almost daily	4 (2%)	0 (0%)	4 (3%)	
	3-5 per week	13 (6%)	0 (0%)	13 (9%)	
	1-2 per week	35 (16%)	5 (8%)	30 (20%)	
	Rarely/never	166 (76%)	61 (92%)	105 (69%)	
Processed Food					<0.001
	1-2 per day	3 (1%)	0 (0%)	3 (2%)	
	3-5 per week	18 (9%)	1 (2%)	17 (12%)	
	1-2 per week	55 (26%)	7 (11%)	48 (33%)	
	Rarely/Never	136 (64%)	58 (88%)	78 (53%)	
	Unknown	6	0	6	

Table S2.

Adherence to food consumption recommended by the Italian guidelines for healthy eating (CREA 2018) for the main food categories calculated including standard school meals to those children who had lunch at school, overall and stratified by age categories.
* Expressed as median (IQR).

		Tot = 218 (%)	6 m – 1 y = 66	2 – 3 yrs = 152	p-value
**Overall Adherence score***		8.00 (6.00 – 9.00)	7.50 (6.00, 8.00)	8.00 (6.00, 9.00)	0.5
**Cereals Adherence**					> 0.9
	Less than GL	2 (1%)	0 (0%)	2 (1%)	
	According to GL	216 (99%)	66 (100%)	150 (99%)	
**Meat Adherence**					0.003
	Less than GL	11 (5%)	8 (12%)	3 (2%)	
	According to GL	14 (6%)	6 (9%)	8 (5%)	
	More than GL	193 (89%)	52 (79%)	141 (93%)	
**Fish Adherence**					0.3
	Less than GL	103 (47%)	26 (39%)	77 (51%)	
	According to GL	107 (49%)	37 (56%)	70 (46%)	
	More than GL	8 (4%)	3 (5%)	5 (3%)	
**Legumes Adherence**					< 0.001
	Less than GL	25 (11%)	12 (18%)	13 (9%)	
	According to GL	157 (72%)	33 (50%)	124 (82%)	
	More than GL	36 (17%)	21 (32%)	15 (10%)	
**Cheeses Adherence**					0.036
	Less than GL	7 (3%)	5 (8%)	2 (1%)	
	According to GL	30 (14%)	11 (17%)	19 (13%)	
	More than GL	181 (83%)	50 (76%)	131 (86%)	
**Vegetables Adherence**					0.6
	Less than GL	45 (21%)	12 (18%)	33 (22%)	
	According to GL	173 (79%)	54 (82%)	119 (78%)	
**Fresh Fruit Adherence**					0.077
	Less than GL	35 (16%)	15 (23%)	20 (13%)	
	According to GL	183 (84%)	44 (67%)	77 (51%)	
**Dried Fruit Adherence**					0.095
	Less than GL	136 (62%)	48 (73%)	88 (58%)	
	According to GL	22 (10%)	6 (9%)	16 (11%)	
	More than GL	60 (28%)	12 (18%)	48 (32%)	
**Milk Adherence**					< 0.001
	Less than GL	136 (62%)	57 (86%)	79 (52%)	
	According to GL	72 (33%)	9 (14%)	63 (41%)	
	More than GL	10 (5%)	0 (0%)	10 (7%)	
**Yogurt Adherence**					0.001
	Less than GL	158 (72%)	57 (86%)	101 (66%)	
	According to GL	37 (17%)	9 (14%)	28 (18%)	
	More than GL	23 (11%)	0 (0%)	23 (15%)	
**Eggs Adherence**					< 0.001
	Less than GL	73 (33%)	40 (61%)	33 (22%)	
	According to GL	97 (44%)	18 (27%)	79 (52%)	
	More than GL	48 (22%)	8 (12%)	40 (26%)	
**Sweets Adherence**					< 0.001
	According to GL	161 (74%)	59 (89%)	102 (67%)	
	More than GL	57 (26%)	7 (11%)	50 (33%)	

**Soft Drinks Adherence**					< 0.001
	According to GL	161 (74%)	59 (89%)	102 (67%)	
	More than GL	57 (26%)	7 (11%)	50 (33%)	
**Processed Food Adherence**					< 0.001
	According to GL	136 (62%)	58 (88%)	78 (51%)	
	More than GL	82 (38%)	8 (12%)	74 (49%)	

Table S3:

Jaccard Index Matrix for adherence clustering for main food categories without accounting for school meals and representing food categories adherence co-occurrence. Co-occurrence was calculated using the Jaccard Similarity index, where 0 represents when 2 food categories adherence never occur together and 1 if they always appear together.

	Cereals	Meat	Fish	Legumes	Cheese	Vegetables	Fruits	Dried_Fruit	Cow_milk	Yogurt	Eggs	Sweets	Sweet_drinks	Processed foods
**Cereals**	1.000	0.159	0.500	0.477	0.302	0.498	0.551	0.104	0.341	0.159	0.231	0.730	0.762	0.621
**Meat**	0.159	1.000	0.161	0.133	0.140	0.169	0.137	0.017	0.124	0.088	0.130	0.158	0.167	0.169
**Fish**	0.500	0.161	1.000	0.352	0.197	0.387	0.425	0.093	0.252	0.161	0.246	0.497	0.460	0.350
**Legumes**	0.477	0.133	0.352	1.000	0.246	0.376	0.405	0.102	0.295	0.189	0.264	0.408	0.405	0.356
**Cheese**	0.302	0.140	0.197	0.246	1.000	0.264	0.258	0.083	0.195	0.128	0.167	0.264	0.277	0.273
**Vegetables**	0.498	0.169	0.387	0.376	0.264	1.000	0.547	0.092	0.216	0.169	0.153	0.494	0.481	0.488
**Fruits**	0.551	0.137	0.425	0.405	0.258	0.547	1.000	0.109	0.261	0.179	0.155	0.533	0.457	0.452
**Dried_Fruit**	0.104	0.017	0.093	0.102	0.083	0.092	0.109	1.000	0.080	0.113	0.125	0.083	0.074	0.090
**Cow_milk**	0.341	0.124	0.252	0.295	0.195	0.216	0.261	0.080	1.000	0.090	0.140	0.287	0.308	0.292
**Yogurt**	0.159	0.088	0.161	0.189	0.128	0.169	0.179	0.113	0.090	1.000	0.160	0.158	0.147	0.146
**Eggs**	0.231	0.130	0.246	0.264	0.167	0.153	0.155	0.125	0.140	0.160	1.000	0.220	0.207	0.177
**Sweets**	0.730	0.158	0.497	0.408	0.264	0.494	0.533	0.083	0.287	0.158	0.220	1.000	0.703	0.659
**Sweet_drinks**	0.762	0.167	0.460	0.405	0.277	0.481	0.457	0.074	0.308	0.147	0.207	0.703	1.000	0.669
**Processed foods**	0.621	0.169	0.350	0.356	0.273	0.488	0.452	0.090	0.292	0.146	0.177	0.659	0.669	1.000

Table S4:

Jaccard Index Matrix for adherence clustering for main food categories accounting for school meals and representing food categories adherence co-occurrence. Co-occurrence was calculated using the Jaccard Similarity index, where 0 represents when 2 food categories adherence never occur together and 1 if they always appear together.

	**Cereals**	**Meat**	**Fish**	**Legumes**	**Cheese**	**Vegetables**	**Fruits**	**Dried_Fruit**	**Cow_milk**	**Yogurt**	**Eggs**	**Sweets**	**Sweet_drinks**	**Processed foods**
**Cereals**	1.000	0.060	0.495	0.711	0.134	0.793	0.839	0.367	0.333	0.161	0.449	0.745	0.769	0.630
**Meat**	0.060	1.000	0.034	0.043	0.100	0.063	0.065	0.067	0.024	0.085	0.037	0.067	0.071	0.095
**Fish**	0.495	0.034	1.000	0.419	0.151	0.451	0.457	0.322	0.252	0.161	0.360	0.497	0.460	0.350
**Legumes**	0.711	0.043	0.419	1.000	0.087	0.642	0.709	0.350	0.316	0.198	0.427	0.551	0.538	0.450
**Cheese**	0.134	0.100	0.151	0.087	1.000	0.140	0.133	0.167	0.085	0.031	0.114	0.165	0.153	0.161
**Vegetables**	0.793	0.063	0.451	0.642	0.140	1.000	0.798	0.371	0.296	0.160	0.459	0.678	0.646	0.601
**Fruits**	0.839	0.065	0.457	0.709	0.133	0.798	1.000	0.387	0.328	0.176	0.466	0.646	0.646	0.556
**Dried_Fruit**	0.367	0.067	0.322	0.350	0.167	0.371	0.387	1.000	0.176	0.202	0.288	0.328	0.292	0.298
**Cow_milk**	0.333	0.024	0.252	0.316	0.085	0.296	0.328	0.176	1.000	0.090	0.363	0.287	0.308	0.292
**Yogurt**	0.161	0.085	0.161	0.198	0.031	0.160	0.176	0.202	0.090	1.000	0.155	0.158	0.147	0.146
**Eggs**	0.449	0.037	0.360	0.427	0.114	0.459	0.466	0.288	0.363	0.155	1.000	0.372	0.363	0.363
**Sweets**	0.745	0.067	0.497	0.551	0.165	0.678	0.646	0.328	0.287	0.158	0.372	1.000	0.703	0.659
**Sweet_drinks**	0.769	0.071	0.460	0.538	0.153	0.646	0.646	0.292	0.308	0.147	0.363	0.703	1.000	0.669
**Processed foods**	0.630	0.095	0.350	0.450	0.161	0.601	0.556	0.298	0.292	0.146	0.363	0.659	0.669	1.000
